# Financial Toxicities Persist for Cancer Survivors Irrespective of Current Cancer Status: An Analysis of Medical Expenditure Panel Survey

**DOI:** 10.1158/2767-9764.CRC-22-0166

**Published:** 2022-10-05

**Authors:** Mohammad A. Karim, Rajesh Talluri, Surendra S. Shastri, Hye-Chung Kum, Sanjay Shete

**Affiliations:** 1 Department of Epidemiology, The University of Texas MD Anderson Cancer Center, Houston, Texas.; 2 Population Informatics Lab, Texas A&M University, College Station, Texas.; 3 Department of Data Science, University of Mississippi Medical Center, Jackson, Mississippi.; 4 Department of Biostatistics, The University of Texas MD Anderson Cancer Center, Houston, Texas.; 5 Department of Health Disparities Research, The University of Texas MD Anderson Cancer Center, Houston, Texas.; 6 Department of Health Policy and Management, School of Public Health, Texas A&M University, College Station, Texas.; 7 Division of Cancer Prevention and Population Science, The University of Texas MD Anderson Cancer Center, Houston, Texas.

## Abstract

This study estimates the out-of-pocket (OOP) expenditures for different cancer types among survivors with current versus no current cancer condition and across sex, which is understudied in the literature. This is a cross-sectional study of Medical Expenditure Panel Survey data for 2009–2018 where the primary outcome was the average per year OOP expenditure incurred by cancer survivors. Of 189,285 respondents, 15,010 (7.93%) were cancer survivors; among them, 46.28% had a current cancer condition. Average per year OOP expenditure for female survivors with a current condition of breast cancer ($1,730), lung cancer ($1,679), colon cancer ($1,595), melanoma ($1,783), non–Hodgkin lymphoma ($1,656), nonmelanoma/other skin cancer (NMSC, $2,118) and two or more cancers ($2,310) were significantly higher than that of women with no history of cancer ($853, all *P* < 0.05). Similarly, average per year OOP expenditure for male survivors with a current condition of prostate cancer ($1,457), lung cancer ($1,131), colon cancer ($1,471), melanoma ($1,474), non–Hodgkin lymphoma ($1,653), NMSC ($1,789), and bladder cancer ($2,157) were significantly higher compared with the men with no history of cancer ($621, all *P* < 0.05). These differences persisted in survivors with no current cancer condition for breast cancer among women; prostate, lung, colon, and bladder cancer among men; and melanoma, NMSC, and two or more cancers among both sexes. OOP expenditure varied across cancer types and by sex for survivors with and without a current cancer condition. These findings highlight the need for targeted interventions for cancer survivors.

**Significance:**

Our study found that OOP expenditures among survivors with a current cancer condition for several cancers were significantly higher than that of individuals without a cancer history. These differences persisted in female with breast cancer; male with prostate, lung, colon, and bladder cancer; and survivors of both sexes with melanoma, and NMSC/other skin cancer, even after there was no current cancer condition.

## Introduction

Cancer is the second leading cause of death in the United States and is projected to cost more than 608,570 lives in 2021 (1). Despite the high mortality associated with cancer, substantial progress has been made against cancer in recent decades (1). With improved treatments and newly discovered drugs, the cancer death rate declined by 31% between 1991 and 2018 (1). Although substantial progress has been made to improve survivorship and reduce mortality associated with cancer, the additional burden of cancer-related financial distress has emerged as a matter of serious concern (2). The financial toxicity of cancer (3) and out-of-pocket (OOP) burden on cancer survivors have garnered considerable attention from researchers, as well as policy makers, in recent times (3–6).

Increasing numbers of cancer survivors are now living longer, sometimes without requiring active treatment while in remission (7). However, long-term survivors may report significant symptom burden (8, 9), posttreatment adverse events such as fatigue and pain (9, 10), and treatment-related late toxicities (11). Moreover, psychologic distress, anxiety, depression, and insomnia are pronounced among long-term cancer survivors (9, 12) and may require additional treatments, resulting in increased costs. Previous studies have examined the costs affecting previously versus recently diagnosed cancer survivors (13–15); however, none of these studies specifically examined OOP costs across cancer types. A 2018 study using 2008–2012 Health and Retirement Study data reported significantly higher total costs for recently diagnosed cancer survivors compared with long-term survivors; however, distinctions were not made across cancer types (13). Similarly, two studies using 2001–2007 and 2008–2010 Medical Expenditure Panel Survey (MEPS) data reported significantly higher OOP expenses among both recently and previously diagnosed cancer survivors compared with noncancer controls, without examining cancer types (14, 15).

The goal of this study is to assess OOP expenditure by cancer status and cancer types across sex. We used the current condition designation of MEPS to stratify cancer survivors between those with a current cancer condition and those with no current cancer condition (16), and examined OOP expenditure by cancer types for these subgroups. We examined OOP expenditure across cancer types because treatment approaches and survival for different cancers vary considerably (7), which may in turn cause variations in OOP expenditure. In addition, we stratified our analysis by sex because, as demonstrated for other health conditions such as diabetes, disease-specific OOP expenditure may vary across sex (17). Adopting a more granular approach compared with the previous studies, we examined average OOP expenditure by current versus no current cancer condition and by specific cancer types across sex, which will help facilitate health policy discussions and formulate better targeted intervention strategies.

## Materials and Methods

### Data Source and Study Sample

Data for our study were obtained from MEPS, a nationally representative survey of the noninstitutionalized U.S. population, which collects information on health care use and expenditure (18). The survey oversamples minority groups and provides person weights in the released public use datasets (19). The MEPS design and data collection process have been described elsewhere (20, 21). For our study, we pooled multiple years of data (2009–2015 and 2018) and adjusted the survey weights accordingly (22). Among several publicly available MEPS data files, we used the Full Year Consolidated file—which provided information on demographics, socioeconomic status, insurance coverage, health status, ever having cancer, and health care expenditure (19)—and the Medical Conditions file, which provided information on select current clinical conditions, including cancer (16). MEPS data were deidentified and publicly available, therefore, our study was exempt from Institutional Review Board approval.

### Definition of Current Cancer Condition, No Current Cancer Condition, and No History of Cancer

In the survey, respondents 18 years or older were asked “Have you ever been told by a doctor or other health professional that you had cancer or a malignancy of any kind?”; those responding yes to this question were then asked “what kind of cancer was it?” (19, 23). On the basis of the responses to these two questions, we identified individuals as cancer survivors and those with no history of cancer.

Information about current conditions was obtained from the Clinical Classifications Software (CCS) codes or CCS Refined (CCSR) codes. Among the cancer survivors, those who had a cancer-specific CCS or CCSR code were identified as (i) survivors with a current cancer condition; the rest of the cancer survivors were identified as (ii) survivors with no current cancer condition. In MEPS, the current condition was defined as “any clinical condition which had an associated health care event or which was being actively experienced by the respondent during the survey year” (16). Thus, respondents with a current cancer condition were those who either had a health care event or reported that they actively experienced cancer in the survey year. Respondents who had CCS or CCSR code for more than one type of cancer or responded that they had history of more than one prior cancer were classified under “two or more cancers” category.

MEPS used International Classification of Diseases, Ninth Revision (ICD-9)-based CCS codes to report current conditions until 2015 and transitioned to ICD-10–based CCSR codes in 2018 (16). Because neither CCS nor CCSR codes were publicly available for the years 2016 and 2017, we were unable to identify cancer cases with a current condition for 2016 and 2017, and excluded these 2 years. Consequently, our final analytic sample consisted of respondents pooled for the years 2009–2015 and 2018. Cases with mismatched cancer types in the survey response and the current condition designation were excluded.

### Primary Outcome Measure

Total OOP expenditure per person per year was the primary outcome variable in our analysis. The total OOP expenditure was the sum of all-cause OOP expenditure incurred per person per year for any health care event, including office-based visits, outpatient visits, emergency room (ER) visits, inpatient stays, prescription medication purchases, home health care events, and other medical equipment and services use (19). All dollar values were inflation adjusted to 2018 U.S. dollars using the consumer price index for Medical Care (24). Expenditure data in MEPS were primarily self-reported with a subset of the responses verified with the health care providers (19). Even with the possibility of underestimation of cost in MEPS (25), as established in previous studies, use of MEPS data enabled us to examine OOP burden of cancer at the national level (14, 26).

### Covariates

The covariates in each of the estimation model were age, cancer types, race/ethnicity, marital status, educational attainment, income level, insurance status, survey year, number of comorbid conditions, and self-reported health status. All covariates except age were categorical variables (Table 1). In the race/ethnicity variable, non-Hispanic White, non-Hispanic Black, and Hispanic were separate categories, while all other race/ethnicities were grouped together into the “Others” category. Marital status was dichotomized to two groups: single (which included individuals who never married or were widowed, divorced, or separated) and married. Educational attainment was categorized into: less than high school diploma, high school diploma, college education or higher, and missing. Income level was categorized into: <200% of federal poverty level, 200% to <400% of federal poverty level, and ≥400% of federal poverty level (27). Insurance status had five categories: private (employer sponsored), private (nonemployer sponsored), Medicaid/dual eligible, Medicare, and Uninsured. The Medicaid/dual eligible category included the individuals who were covered by both Medicaid and Medicare.

**TABLE 1:** Sociodemographic characteristics of adult U.S. population with no history of cancer and cancer survivors with a current cancer condition and no current cancer condition, 2009–2015 and 2018

	Women									Men								
	No history of cancer (raw *n* = 92,437)			Current cancer condition (raw *n* = 3,716)			No current cancer condition (raw *n* = 5,186)			No history of cancer (raw *n* = 81,838)			Current cancer condition (raw *n* = 3,230)			No current cancer condition (raw *n* = 2,878)		
Variable	Raw *n*	Weighted *n*	Weighted %	Raw *n*	Weighted *n*	Weighted %	Raw *n*	Weighted *n*	Weighted %	Raw *n*	Weighted *n*	Weighted %	Raw *n*	Weighted *n*	Weighted %	Raw *n*	Weighted *n*	Weighted %
**Cancer status and type**																		
No history of cancer	92,437	107,371,495	100.00	NA	NA	NA	NA	NA	NA	81,838	103,218,167	100.00	NA	NA	NA	NA	NA	NA
Cervical cancer	NA	NA	NA	150	161,247	2.97	847	1,068,884	14.28	NA	NA	NA	NA	NA	NA	NA	NA	NA
Breast cancer	NA	NA	NA	1,271	1,786,934	32.95	972	1,405,370	18.77	NA	NA	NA	NA	NA	NA	NA	NA	NA
Prostate cancer	NA	NA	NA	NA	NA	NA	NA	NA	NA	NA	NA	NA	892	1,230,541	24.16	672	976,012	21.09
Lung cancer	NA	NA	NA	99	131,137	2.42	39	43,084	0.58	NA	NA	NA	99	126,382	2.48	39	55,176	1.19
Colon cancer	NA	NA	NA	134	151,486	2.79	199	253,695	3.39	NA	NA	NA	163	218,424	4.29	191	263,248	5.69
Melanoma	NA	NA	NA	149	270,819	4.99	295	507,968	6.78	NA	NA	NA	166	295,379	5.80	248	461,994	9.98
Non–Hodgkin lymphoma	NA	NA	NA	83	119,854	2.21	64	95,426	1.27	NA	NA	NA	83	134,315	2.64	69	103,116	2.23
Nonmelanoma/ other skin cancer	NA	NA	NA	724	1,300,171	23.97	1,181	2,127,129	28.41	NA	NA	NA	898	1,617,101	31.75	981	1,726,733	37.31
Bladder cancer	NA	NA	NA	38	48,612	0.90	23	33,447	0.45	NA	NA	NA	104	179,228	3.52	62	93,110	2.01
Other/ unspecified	NA	NA	NA	851	1,130,565	20.85	1,356	1,658,538	22.15	NA	NA	NA	608	926,017	18.18	526	798,044	17.24
≥ Two cancers	NA	NA	NA	217	322,232	5.94	210	293,971	3.93	NA	NA	NA	217	366,050	7.19	90	150,533	3.25
**Race/ethnicity**																		
Non-Hispanic White	36,805	67,006,708	62.41	2,484	4,456,563	82.18	3,622	6,320,396	84.41	34,777	65,120,015	63.09	2,336	4,365,836	85.71	2,210	4,043,615	87.37
Non-Hispanic Black	20,168	14,273,103	13.29	519	374,965	6.91	651	435,528	5.82	14,699	11,908,462	11.54	468	352,109	6.91	311	238,988	5.16
Hispanic	25,959	16,859,054	15.70	495	367,796	6.78	667	474,097	6.33	23,649	17,621,623	17.07	288	232,927	4.57	259	234,196	5.06
Others	9,505	9,232,630	8.60	218	223,735	4.13	246	257,492	3.44	8,713	8,568,067	8.30	138	142,565	2.80	98	111,167	2.40
**Marital status**																		
Single^a^	50,269	52,977,458	49.34	1,865	2,462,367	45.41	2,869	3,779,027	50.47	39,927	49,057,238	47.53	1,044	1,499,100	29.43	1,043	160,4065	34.66
Married	42,168	54,394,037	50.66	1,851	2,960,691	54.59	2,317	3,708,486	49.53	41,911	54,160,928	52.47	2,186	3,594,337	70.57	1,835	302,3901	65.34
**Educational attainment**																		
< High school diploma	18,137	13,981,852	13.02	536	542,368	10.00	834	841,358	11.24	16,775	14,706,007	14.25	500	527,786	10.36	405	481,082	10.40
High school diploma	33,485	38,078,908	35.46	1,452	2,052,912	37.86	2,114	2,988,776	39.92	30,792	39,191,175	37.97	1,163	1,807,971	35.50	1,033	156,9773	33.92
≥ College education	33,566	47,822,870	44.54	1,479	2,430,228	44.81	1,909	3,169,802	42.33	27,720	42,109,400	40.80	1,371	2,462,559	48.35	1,249	225,6183	48.75
Missing	7,249	7,487,865	6.97	249	397,550	7.33	329	487,577	6.51	6,551	7,211,584	6.99	196	295,121	5.79	191	320,927	6.93
**Income level**																		
<200% of federal poverty level	41,495	35,260,388	32.84	1,370	1,592,859	29.37	2,121	2,450,842	32.73	30,005	28,462,518	27.58	1,026	1,220,191	23.96	866	1,133,669	24.50
200% to <400% of federal poverty level	26,579	31,742,313	29.56	1,052	1,493,071	27.53	1,488	2,117,417	28.28	25,853	31,372,342	30.39	859	1,244,921	24.44	787	1,170,785	25.30
≥400% of federal poverty level	24,363	40,368,794	37.60	1,294	2,337,128	43.10	1,577	2,919,253	38.99	25,980	43,383,306	42.03	1,345	2,628,325	51.60	1,225	2,323,512	50.21
**Insurance status**																		
Private (employer sponsored)	44,042	61,143,226	56.95	1,728	2,759,370	50.88	2,142	3,514,924	46.94	41,839	60,785,918	58.89	1,412	2,486,515	48.82	1,367	2,385,802	51.55
Private (nonemployer sponsored)	7,639	11,184,108	10.42	531	916,883	16.91	698	1,153,696	15.41	6,534	9,976,135	9.67	569	970,337	19.05	450	770,487	16.65
Medicaid/dual eligible	18,273	14,259,959	13.28	618	565,611	10.43	921	898,364	12.00	9,696	8,718,953	8.45	362	345,920	6.79	248	264,938	5.72
Medicare	6,585	8,131,888	7.57	698	1,030,552	19.00	980	1,425,841	19.04	4,946	6,176,535	5.98	808	1 ,179,316	23.15	670	1,024,278	22.13
Uninsured	15,898	12,652,313	11.78	141	150,641	2.78	445	494,688	6.61	18,823	17,560,626	17.01	79	111,348	2.19	143	182,460	3.94
**Survey year**																		
2009	11,928	12,885,970	12.00	461	644,034	11.88	667	918,562	12.27	10,476	12,533,478	12.14	410	632,270	12.41	309	467,412	10.10
2010	10,752	13,019,930	12.13	430	613,064	11.30	604	937,259	12.52	9,502	12,652,110	12.26	352	565,708	11.11	316	510,220	11.02
2011	11,569	13,257,408	12.35	482	706,518	13.03	605	832,032	11.11	10,208	12,642,315	12.25	409	648,623	12.73	343	551,007	11.91
2012	12,789	13,426,056	12.50	521	738,766	13.62	658	869,926	11.62	11,401	12,800,383	12.40	416	607,623	11.93	375	580,938	12.55
2013	12,077	13,453,874	12.53	455	676,793	12.48	605	960,243	12.82	10,718	12,929,583	12.53	360	596,691	11.71	354	642,027	13.87
2014	11,411	13,572,148	12.64	431	693,692	12.79	616	953,366	12.73	10,096	13,068,864	12.66	385	701,512	13.77	320	562,359	12.15
2015	11,691	13,701,065	12.76	461	709,879	13.09	657	948,653	12.67	10,462	13,207,538	12.80	434	713,102	14.00	357	593,829	12.83
2018	10,220	14,055,044	13.09	475	640,312	11.81	774	1,067,471	14.26	8,975	13,383,895	12.97	464	627,908	12.33	504	720,174	15.56
**Number of comorbid conditions**																		
Zero	37,082	41,266,220	38.43	463	635,535	11.72	686	1,036,269	13.84	34,072	39,686,930	38.45	243	338,627	6.65	304	490,832	10.61
One	19,589	23,438,807	21.83	549	815,282	15.03	813	1,169,529	15.62	18,877	24,652,945	23.88	388	644,884	12.66	431	741,033	16.01
Two	12,780	15,595,161	14.52	646	985,335	18.17	899	1,313,735	17.55	11,689	15,829,467	15.34	630	1,023,200	20.09	527	845,984	18.28
Three	9,382	11,465,780	10.68	647	987,588	18.21	903	1,355,629	18.11	7,744	10,537,546	10.21	629	953,679	18.72	565	880,799	19.03
≥Four	13,604	15,605,527	14.53	1,411	1,999,317	36.87	1,885	2,612,350	34.89	9,456	12,511,279	12.12	1,340	2,133,047	41.88	1,051	1,669,318	36.07
**Health status**																		
Fair/poor	13,988	13,648,439	12.71	1071	1,310,864	24.17	1,287	1,595,559	21.31	10,120	11,358,664	11.00	1,005	1,391,303	27.32	633	939,360	20.30
Good	28,319	30,347,707	28.26	1,169	1,685,116	31.07	1,656	2,268,204	30.29	23,425	27,903,465	27.03	1,037	1,654,293	32.48	928	1,419,364	30.67
Very good/ excellent	50,130	63,375,348	59.02	1,476	2,427,078	44.75	2,243	3,623,750	48.40	48,293	63,956,038	61.96	1,188	2,047,840	40.21	1,317	2,269,241	49.03

Abbreviation: NA, not applicable.

^a^The “single” category includes individuals who never married or were widowed, divorced, or separated.

### Statistical Analysis

Because our data consist of three types of participants (i) those without cancer (ii) survivors with current cancer condition, and (iii) survivors with no current cancer condition, a substantial number of survey respondents had zero OOP expenditure. To account for the heterogeneous samples and zero inflation, we adopted a two-part regression model. We used logistic regression as the first part to model the probability of incurring any expenditure and used generalized linear model regression with log link and gamma distribution as the second part to model the nonzero expenditure (28). This technique was used in the second part because the gamma distribution models the non-negative and right-skewed expenditure data appropriately, whereas the log link helps avoid retransformation (28). Also, we stratified the analyses by those with current cancers and those with no current cancer. Within each analysis, types of cancer were used as covariate.

We estimated the adjusted average OOP expenditure for several cancer types (29). A permutation test was used to estimate the *P* values for the OOP expenditure difference for each category compared with the “no history of cancer” reference category (30). The permutation test is a nonparametric method which allowed us to construct the empirical null distribution of the incremental mean values for each cancer category with respect to the “no history of cancer” reference. *P* values represent the statistical significance obtained using 1,000 permutated replicates to test the hypothesis that the estimated average OOP expenditure for each cancer category is different than the “no history of cancer” category (two-sided *P* value; ref. 30). We conducted the permutation test in several steps. First, we permuted the outcome variable (i.e., OOP expenditure) 1,000 times. Then we estimated the average OOP expenditure for all 1,000 permutated outcome variables by applying the two-part model, which formed the empirical null distribution. Finally, two-sided *P* values were obtained by comparing the estimated OOP expenditure from the actual data with the empirical null distribution generated through permutation. We conducted analyses by stratifying our sample by “current cancer condition” and “no current cancer condition” status. All analyses for male and female survivors were conducted separately.

To compare the differences in cancer-attributable OOP expenditure between female and male survivors, we first subtracted the estimated OOP expenditure for the “no history of cancer” category from each cancer type for women and men separately. Subtracting these cancer-attributable OOP expenditure values for men from the respective values for women yielded the incremental cancer-attributable OOP expenditures for each cancer type. The *P* values were obtained by comparing these differences in estimated OOP expenditures between women and men to the respective differences in 1,000 replicate data.

All analyses were conducted in SAS 9.4 (SAS Institute; RRID:SCR_008567) and Stata15 (StataCorp; RRID:SCR_012763) software, and two-sided *P* < 0.05 was considered statistically significant. We incorporated survey weights in all our descriptive and covariate adjusted analyses and employed survey specific commands (i.e., svyset and svy: prefix) in Stata.

### Data Availability

MEPS data analyzed in this study are publicly available from the Agency for Healthcare Research and Quality website at: https://meps.ahrq.gov/data_stats/download_data_files.jsp.

## Results

### Characteristics of The Study Sample

Our study sample included 189,285 adult individuals (weighted *N* = 233,221,635) with an average age of 46.61 years. The weighted percentage of non-Hispanic White, non-Hispanic Black, Hispanic, and other race/ethnicity was 64.88%, 11.83%, 15.35%, and 7.95%, respectively. The study sample included 15,010 cancer survivors (weighted *n* = 22,631,973) with average age of 63.99 years. Among the cancer survivors, the weighted percentage of non-Hispanic White, non-Hispanic Black, Hispanic, and other race/ethnicity was 84.78%, 6.19%, 5.78%, and 3.25%, respectively. Of the cancer survivors, 10.57% (weighted percentage) did not have any high school diploma and 28.27% lived below 200% of the federal poverty level.

The average age of the female cancer survivors (62.44 years) was lower than the average age of the male survivors (66.06 years). Among the 8,902 (weighted *n* = 12,910,571) female survivors, 42% had a current cancer condition; among the 6,108 (weighted *n* = 9,721,402) male survivors, 52.39% had a current cancer condition.

Table 1 illustrates the sociodemographic characteristics of the study sample stratified by sex and cancer status (i.e., no history of cancer, current cancer condition, no current cancer condition). The percentage of non-Hispanic White respondents was similar between survivors with a current cancer condition (female 82.18%, male 85.71%) and survivors with no current cancer condition (female 84.41%, male 87.37%), whereas it was lower among those with no history of cancer (female 62.41%, male 63.09%). There was no substantial difference in educational attainment between respondents with a current cancer condition versus no current cancer condition among either female or male cancer survivors. Most cancer survivors had income ≥400% of the federal poverty level, with a higher percentage of male survivors (current cancer condition 51.60%, no current cancer condition 50.21%) in this category compared with female survivors (current cancer condition 43.10%, no current cancer condition 38.99%). Although the uninsured rate was similar among female and male survivors with a current cancer condition, among the survivors with no current cancer condition, more women (6.61%) were uninsured than men (3.94%).

### Estimated OOP Expenditure Among Female Cancer Survivors

Among female cancer survivors with a current cancer condition, those with breast cancer ($1,730, *P* < 0.001), lung cancer ($1,679, *P* = 0.009), colon cancer ($1,595, *P* = 0.010), melanoma ($1,783, *P* = 0.002), non–Hodgkin lymphoma ($1,656, *P* = 0.018), nonmelanoma skin cancer (NMSC)/other skin cancer ($2,118, *P* < 0.001), and two or more cancers ($2,310, *P* < 0.001) had statistically significantly higher OOP expenditures compared with the females with no history of cancer ($853); however, the difference was not statistically significant for females with a current cervical cancer condition ($882, *P* = 0.855; Table 2).

**TABLE 2:** Average per year OOP expenditure for female cancer survivors^a^

	Current cancer condition		No current cancer condition	
Cancer status and type	Average out-of-pocket expenditures, $US^b^	*P* ^c^	Average out-of-pocket expenditures, $US^b^	*P* ^c^
No history of cancer [Reference]	853		857	
Cervical cancer	882	0.855	1,207	0.007
Breast cancer	1,730	<0.001	1,364	<0.001
Lung cancer	1,679	0.009	1,131^d^	0.322
Colon cancer	1,595	0.010	1,142	0.083
Melanoma	1,783	0.002	1,396	0.015
Non–Hodgkin lymphoma	1,656	0.018	1,216	0.126
Nonmelanoma/other skin cancer	2,118	<0.001	1,506	<0.001
Bladder cancer	1,848^d^	0.024	1,015^d^	0.650
Other/unspecified	1,621	<0.001	1,106	0.008
≥Two cancers	2,310	<0.001	1,578	0.007

^a^Estimates were obtained from survey weighted and covariate-adjusted analysis of pooled Medical Expenditure Panel Survey data for the years 2009–2015 and 2018. The dollar values were inflation-adjusted to 2018 U.S. dollars using the consumer price index (CPI).

^b^Estimated average OOP expenditure by applying the two-part regression model to specific cancer subtypes. Each model was adjusted for age, cancer status and type, race/ethnicity, marital status, educational attainment, income level, insurance status, survey year, number of comorbid conditions, and self-reported health status.

^c^ *P* value represents the statistical significance obtained using 1,000 permutated replicates to test the hypothesis that the estimated average OOP expenditure for each cancer category is different than the “no history of cancer” category (two-sided *P* value). Each replicate model was adjusted for the same set of predictors as the base model, and the dependent variable, OOP expenditure, was permuted for each replicate analysis.

^d^Unweighted sample size less than 60.

Among female cancer survivors with no current cancer condition, those with cervical cancer ($1,207, *P* = 0.007), breast cancer ($1,364, *P* < 0.001), melanoma ($1,396, *P* = 0.015), NMSC/other skin cancer ($1,506, *P* < 0.001), and two or more cancers ($1,578, *P* = 0.007) had significantly higher OOP expenditures compared with the females with no history of cancer ($857; Table 2).

### Estimated OOP Expenditure Among Male Cancer Survivors

Among male cancer survivors with a current cancer condition, those with prostate cancer ($1,457, *P* < 0.001), lung cancer ($1,131, *P* = 0.027), colon cancer ($1,471, *P* = 0.001), melanoma ($1,474, *P* < 0.001), non–Hodgkin lymphoma ($1,653, *P* = 0.005), NMSC/other skin cancer ($1,789, *P* < 0.001), bladder cancer ($2,157, *P* < 0.001), and two or more cancers ($2,641, *P* < 0.001) had statistically significantly higher OOP expenditures than men with no history of cancer ($621; Table 3).

**TABLE 3:** Average per year OOP expenditures for male cancer survivors^a^

	Current cancer condition		No current cancer condition	
Cancer status and type	Average out-of-pocket expenditures, $US^b^	*P* ^c^	Average out-of-pocket expenditures, $US^b^	*P* ^c^
No history of cancer [Reference]	621		621	
Prostate cancer	1,457	<0.001	1,152	0.002
Lung cancer	1,131	0.027	1,323^d^	0.031
Colon cancer	1,471	0.001	966	0.028
Melanoma	1,474	<0.001	1,351	0.001
Non–Hodgkin lymphoma	1,653	0.005	646	0.916
Nonmelanoma/other skin cancer	1,789	<0.001	1,478	<0.001
Bladder cancer	2,157	<0.001	1,321	0.019
Other/unspecified	2,255	<0.001	1,080	0.003
≥ Two cancers	2,642	<0.001	1,433	0.009

^a^Estimates were obtained from survey weighted and covariate adjusted analysis of pooled Medical Expenditure Panel Survey data for the years 2009–2015 and 2018. The dollar values were inflation-adjusted to 2018 U.S. dollars using the consumer price index (CPI).

^b^Estimated average OOP expenditure by applying the two-part regression model to specific cancer subtypes. Each model was adjusted for age, cancer status and types, race/ethnicity, marital status, educational attainment, income level, insurance status, survey year, number of comorbid conditions, and self-reported health status.

^c^ *P* value represents the statistical significance obtained using 1,000 permutated replicates to test the hypothesis that the estimated average OOP expenditure for each cancer category is different than “no history of cancer” category (two-sided *P*-value). Each replicate model was adjusted for the same set of predictors as the base model, and the dependent variable, OOP expenditure, was permuted for each replicate analysis.

^d^Unweighted sample size less than 60.

Among male cancer survivors with no current cancer condition, those with prostate cancer ($1,152, *P* = 0.002), colon cancer ($966, *P* = 0.028), melanoma ($1,351, *P* < 0.001), NMSC/other skin cancer ($1,478, *P* < 0.001), bladder cancer ($1,321, *P* = 0.019), and two or more cancers ($1,433, *P* = 0.009) had significantly higher OOP expenditures compared with men with no history of cancer ($621; Table 3).

### Differences in Cancer-attributable OOP Expenditures Among Female Cancer Survivors Compared with Male Cancer Survivors

Table 4 shows incremental cancer-attributable OOP expenditures for female cancer survivors compared with male cancer survivors. Among survivors with current cancer condition, cancer-attributable OOP expenditures for females with two or more cancers was significantly lower than for males with two or more cancers (difference in cancer attributable OOP = −$564, *P* = 0.021). Among cancer survivors with no current cancer condition, cancer-attributable OOP expenditures for females with NMSC/other skin cancer was significantly lower than for males with NMSC/other skin cancer (difference in cancer attributable OOP = −$208, *P* = 0.044; Table 4).

**TABLE 4:** Incremental cancer-attributable per-year OOP expenditures for female cancer survivors compared with male cancer survivors

	Current cancer condition		No current cancer condition	
Cancer status and type	Incremental cancer-attributable average out-of-pocket expenditure, $US^a^	*P* ^b^	Incremental cancer-attributable average out-of-pocket expenditure, $US^a^	*P* ^b^
Lung cancer	316	0.236	−428	0.264
Colon cancer	−108	0.638	−60	0.765
Melanoma	77	0.693	−191	0.263
Non–Hodgkin lymphoma	−229	0.368	334	0.271
Nonmelanoma/other skin cancer	97	0.364	−208	0.044
Bladder cancer	−541	0.119	−542	0.180
Other/unspecified	−866	<0.001	−210	0.061
≥ Two cancers	−564	0.021	−91	0.669

^a^Cancer-attributable incremental per year OOP expenditure values were obtained by deducting cancer attributable OOP expenditure values for males from the respective values for females for each cancer type. Negative sing means that the cancer-attributable OOP expenditure for females was lower than for males.

^b^ *P* value represents the statistical significance obtained using 1,000 permutated replicates to test the hypothesis that for each specific cancer type the cancer attributable average OOP expenditures for female survivors are different from those of male survivors.

## Discussion

In this nationally representative study, we estimated average total per year OOP expenditures for several common cancer types among survivors with current and no current cancer conditions. Our results show that the OOP expenditures among survivors with a current cancer condition of breast cancer (female only), prostate cancer (male only), lung cancer, colon cancer, melanoma, non–Hodgkin lymphoma, NMSC/other skin cancer, bladder cancer, and two or more cancers were significantly higher than the OOP expenditures among individuals with no history of cancer of respective sex. These differences were observed in female survivors with breast cancer; male survivors with prostate, lung, colon, and bladder cancer; and survivors of both sexes with melanoma, NMSC/other skin cancer, and two or more cancers even when survivors had no current cancer condition. Among women with cervical cancer, average OOP expenditure was not significantly higher for those with a current cancer condition compared to women with no history of cancer; however, it was higher for those with no current cancer condition compared with women with no history of cancer.

As expected, we observed higher OOP expenditure among survivors with a current cancer condition compared with those with no current cancer for most cancer types (except cervical cancer). The higher OOP estimates are likely attributable to the greater health care needs among individuals recently diagnosed with cancer (31, 32). Cancer treatment incurs its highest costs in the initial and terminal phases of care, and the cost is usually lower in the continuing phase. In addition to cancer-related care, additional health service needs, such as home health care and mental health care, are elevated among recently diagnosed survivors. According to Chesney and colleagues, home health care is utilized by 43.7% of elderly cancer survivors in the first month after surgery, and the percentage decreases to 12.6% 5 years after surgery (33). The initial treatment cost, coupled with the elevated supportive health care needs, may have resulted in the higher OOP expenditures reported in our study among those with a current cancer condition.

One notable finding in our study is that the OOP expenditures for survivors with no current cancer condition for several cancer types (breast cancer among women; prostate, lung, colon, and bladder cancers among men; and melanoma, NMSC/other skin cancer, and two or more cancers among both sexes) were significantly higher compared to those with no cancer history. This finding highlights the persistence of higher health care spending among survivors who do not currently experience cancer or actively receive treatment for cancer. Although the maintenance phase of cancer care may incur lower costs than the initial phase (31), long-term cancer survivors may still experience heightened health needs due to several persistent psychologic and physiologic conditions. Compared with the general population, significantly higher depression and anxiety have been reported among younger (<60 years) long-term cancer survivors (34). Although the literature on depression and anxiety related to OOP burden in cancer survivors is lacking, total financial burden is well reported (35, 36). Diagnosis of depression results in around 32% higher total expenditures among cancer survivors (35), which may be associated with higher OOP expenditure. In addition to mental health care, supportive services such as home health care are used by more than 12% of long-term cancer survivors (33). The persistent mental and home health care needs are possible reasons for the higher OOP expenditures among long-term survivors with no current cancer condition.

An interesting finding in our study was that OOP expenditure among women with a current cervical cancer condition was not significantly higher than women without a history of cancer. A possible explanation for this finding may be the way the treatment-related cost is transferred to the survivors by the insurers. Although extensive treatment may be required for cervical cancer (37, 38), the insurers transfer only a fraction of the total treatment-related costs to the patients (39). Blanco and colleagues reported that the median OOP cost for commercially insured women with cervical cancer in the first 12 months after diagnosis was $2,253, which was only 3.9% of the total treatment-related cost (39). This lower cost transfer to recently diagnosed patients with cervical cancer may help explain the reduced OOP burden on this subgroup.

In contrast, we found that OOP expenditure for cervical cancer survivors with no current cancer was significantly higher than the OOP expenditure for women without a cancer history. Substantial physiologic and psychologic needs (40–43) demonstrated by long-term cervical cancer survivors may explain this finding. Long-term cervical cancer survivors experience several physiologic issues, many of which are associated with treatment interventions in the pelvic region (40). Treatment-related adverse events include bladder dysfunction, gastrointestinal complications, sexual dysfunction, and lymphedema (40–43). Bladder symptoms are very common, with 96.2% of cervical and endometrial cancer survivors reporting bladder storage issues and 82.7% reporting incontinence issues 1 year after treatment, and these percentages are significantly higher than in people with no cancer history (40, 41). In addition, lymphedema, chronic radiation proctitis with late onset, and sexual dysfunction may affect long-term cervical cancer survivors (40, 42). The clinical care related to these physiologic conditions is the most likely cause of higher OOP burden observed in this subgroup. This finding underscores the need to provide financial support to cervical cancer survivors even when they are a few years removed from their cancer diagnosis.

Similar to cervical cancer, lymphedema is observed in more than 40% of breast cancer survivors and in lower percentages among several other cancers (44). In addition, chronic radiation proctitis is observed in prostate, urinary bladder, uterine, and anal cancers, where radiotherapy poses a risk of rectum injury (45). In our study, some of these cancer types, namely breast cancer (women) and prostate and bladder cancers (men), among survivors with no current cancer condition demonstrated significantly higher OOP expenditures compared with individuals without a cancer history. This is an indication that the long-term adverse effects related to cancer treatment may prevent cancer survivors’ OOP costs from returning to their precancer level. Specific aspects of these long-term adverse effects causing higher OOP costs should be investigated further in future research.

In addition to total OOP expenses incurred by female and male survivors separately, we investigated incremental cancer-attributable costs for female survivors compared with male survivors. We observed significantly lower cancer-attributable OOP expenditures for female survivors only among those with two or more cancers (among survivors with current cancer) and NMSC/other skin cancer (among survivors with no current cancer). These results suggest that sex does not play a significant role in OOP expenditure variations across most cancer types.

### Significance of Findings and/or Policy Implications

Cancer is physically and psychologically debilitating, and it reduces survivor's ability and engagement to work. Considering this aspect of cancer, it is vitally important to adopt policy actions targeting the most vulnerable survivors. A good example of a federal initiative to alleviate financial distress related to cancer screening and diagnosis is the National Breast and Cervical Cancer Early Detection Program (NBCCEDP). This program reduces financial barriers related to breast and cervical cancer screening and diagnosis among underserved women and provides Medicaid access after diagnosis. Public health interventions similar to the NBCCEDP for other cancer types could lessen the financial burden on a substantial number of cancer survivors. To implement such interventions for other cancers in a cost-efficient manner, providing targeted assistance to the individuals with most in need is of vital importance. Targeting long-term survivors with financial intervention is equally important as targeting the survivors with current cancer because both groups may experience high OOP burden depending on the cancer type. To that extent, our study reports the specific cancer types with high OOP expenditures among survivors with current and no current cancer across both sexes, which may help identify the most financially vulnerable cancer survivors.

### Strengths and Limitations

Our study was conducted using nationally representative data, which is a strength of this study. Despite this strength of our study, there are a few limitations. First, although based on nationally representative general adult population, the study may not be representative of cancer survivors because survey participants are usually a self-selected group in the general population and they are generally healthier than the nonparticipants (46). The self-reported nature of MEPS carries a possibility of recall bias (47); however, possibilities of self-selection bias prevalent in web surveys (48) is potentially reduced through the implementation of personal interviews (49) with a population-representative complex survey design in MEPS. Second, the cost amounts might also be underestimated in MEPS due to the self-reported nature of the survey (50, 51); however, a subset of the responses were verified by MEPS with health care providers data (19). Third, we were unable to incorporate cancer-related clinical information (e.g., age at diagnosis, time since diagnosis, stage) in our analyses due to the unavailability of those variables in MEPS. Finally, we had to exclude 2016 and 2017 data because CCS codes were not available for those years. Despite these limitations, MEPS is a valuable data source because it is the only nationally representative survey that collects health care utilization and expenditure data in the United States (50–52).

## Conclusion

We estimated the OOP expenditures for female and male survivors for several cancer types across current versus no current cancer conditions. Financial distress affects all aspects of life for cancer survivors, from negatively affecting the purchase of basic necessities like food to contributing as a risk factor for mortality (53). Amid an ongoing discussion on financial toxicity of cancer, several interventions to alleviate the OOP burden on the survivors have been suggested (54, 55). Our study highlights that the financial distress varies across cancer types among the survivors with and without a current cancer condition. This highlights the need for targeted intervention to alleviate the burden on most financially vulnerable cancer survivors. Our findings will inform policy discussions around the financial toxicity of cancer and help formulate targeted interventions.

## References

[1] Siegel RL , Miller KD, Fuchs HE, Jemal A. Cancer statistics, 2021. CA Cancer J Clin 2021;71:7–33. 33433946 10.3322/caac.21654

[2] Zafar SY , Peppercorn JM, Schrag D, Taylor DH, Goetzinger AM, Zhong X, . The financial toxicity of cancer treatment: a pilot study assessing out-of-pocket expenses and the insured cancer patient's experience. Oncologist 2013;18:381–90. 23442307 10.1634/theoncologist.2012-0279 PMC3639525

[3] Carrera PM , Kantarjian HM, Blinder VS. The financial burden and distress of patients with cancer: understanding and stepping-up action on the financial toxicity of cancer treatment. CA Cancer J Clin 2018;68:153–65. 29338071 10.3322/caac.21443 PMC6652174

[4] Chino F , Peppercorn JM, Rushing C, Kamal AH, Altomare I, Samsa G, . Out-of-pocket costs, financial distress, and underinsurance in cancer care. JAMA Oncol 2017;3:1582–4. 28796862 10.1001/jamaoncol.2017.2148 PMC5824215

[5] Karim MA , Singal AG, Ohsfeldt RL, Morrisey MA, Kum H-C. Health services utilization, out-of-pocket expenditure, and underinsurance among insured non-elderly cancer survivors in the United States, 2011–2015. Cancer Med 2021;10:5513–23. 34327859 10.1002/cam4.4103 PMC8366084

[6] Ekwueme DU , Zhao J, Rim SH, de Moor JS, Zheng Z, Khushalani JS, . Annual out-of-pocket expenditures and financial hardship among cancer survivors aged 18–64 years - United States, 2011–2016. MMWR Morb Mortal Wkly Rep 2019;68:494–9. 31170127 10.15585/mmwr.mm6822a2 PMC6553808

[7] Miller KD , Nogueira L, Mariotto AB, Rowland JH, Yabroff KR, Alfano CM, . Cancer treatment and survivorship statistics, 2019. CA Cancer J Clin 2019;69:363–85. 31184787 10.3322/caac.21565

[8] Bernat JK , Wittman DA, Hawley ST, Hamstra DA, Helfand AM, Haggstrom DA, . Symptom burden and information needs in prostate cancer survivors: a case for tailored long-term survivorship care. BJU Int 2016;118:372–8. 26389529 10.1111/bju.13329 PMC4835263

[9] Arndt V , Koch-Gallenkamp L, Jansen L, Bertram H, Eberle A, Holleczek B, . Quality of life in long-term and very long-term cancer survivors versus population controls in Germany. Acta Oncol 2017;56:190–7. 28055266 10.1080/0284186X.2016.1266089

[10] Cramer JD , Johnson JT, Nilsen ML. Pain in head and neck cancer survivors: prevalence, predictors, and quality-of-life impact. Otolaryngol Head Neck Surg 2018;159:853–8. 29943677 10.1177/0194599818783964

[11] McDowell LJ , Rock K, Xu W, Chan B, Waldron J, Lu L, . Long-term late toxicity, quality of life, and emotional distress in patients with nasopharyngeal carcinoma treated with intensity modulated radiation therapy. Int J Radiat Oncol Biol Phys 2018;102:340–52. 30191868 10.1016/j.ijrobp.2018.05.060

[12] Yi JC , Syrjala KL. Anxiety and depression in cancer survivors. Med Clin North Am 2017;101:1099–113. 28992857 10.1016/j.mcna.2017.06.005 PMC5915316

[13] Sullivan J , Thornton Snider J, van Eijndhoven E, Okoro T, Batt K, DeLeire T. The well-being of long-term cancer survivors. Am J Manag Care 2018;24:188–95. 29668209

[14] Guy GP Jr , Ekwueme DU, Yabroff KR, Dowling EC, Li C, Rodriguez JL, . Economic burden of cancer survivorship among adults in the United States. J Clin Oncol 2013;31:3749–57. 24043731 10.1200/JCO.2013.49.1241 PMC3795887

[15] Short PF , Moran JR, Punekar R. Medical expenditures of adult cancer survivors aged <65 years in the United States. Cancer 2011;117:2791–800. 21656757 10.1002/cncr.25835 PMC4124459

[16] Agency for Healthcare Research and Quality. MEPS HC-207: 2018 medical conditions; 2020. Available from: https://meps.ahrq.gov/data_stats/download_data/pufs/h207/h207doc.shtml.

[17] Williams JS , Bishu K, Dismuke CE, Egede LE. Sex differences in healthcare expenditures among adults with diabetes: evidence from the medical expenditure panel survey, 2002–2011. BMC Health Serv Res 2017;17:259. 28399859 10.1186/s12913-017-2178-3 PMC5387347

[18] Cohen JW , Cohen SB, Banthin JS. The medical expenditure panel survey: a national information resource to support healthcare cost research and inform policy and practice. Med Care 2009;47:S44–50. 19536015 10.1097/MLR.0b013e3181a23e3a

[19] Agency for Healthcare Research and Quality. MEPS HC-209: 2018 full year consolidated data file; 2020. Available from: https://meps.ahrq.gov/data_stats/download_data/pufs/h209/h209doc.shtml.

[20] Research AH and Quality. MEPS-HC panel design and collection process; 2021. Available from: https://www.meps.ahrq.gov/survey_comp/hc_data_collection.jsp.

[21] Chowdhury SR , Machlin SR, Gwet KL. Methodology report #33: sample designs of the medical expenditure panel survey household component, 1996–2006 and 2007–2016; 2021. Available from: https://meps.ahrq.gov/data_files/publications/mr33/mr33.shtml.

[22] Agency for Healthcare Research and Quality. MEPS HC-036: 1996–2018 pooled linkage file for common variance structure; 2020. Available from: https://www.meps.ahrq.gov/data_stats/download_data/pufs/h36/h36u18doc.pdf.

[23] Research AH, MEPS Q. Priority condition enumeration (PE) Section - 2018; 2021. Available from: https://meps.ahrq.gov/survey_comp/hc_survey/2018/PE-2018.pdf.

[24] Dunn A , Grosse SD, Zuvekas SH. Adjusting health expenditures for inflation: a review of measures for health services research in the United States. Health Serv Res 2018;53:175–96. 27873305 10.1111/1475-6773.12612 PMC5785315

[25] Aizcorbe A , Liebman E, Pack S, Cutler DM, Chernew ME, Rosen AB. Measuring health care costs of individuals with employer-sponsored health insurance in the U.S.: a comparison of survey and claims data. Stat J IAOS 2012;28:43–51. 26146526 10.3233/SJI-2012-0743 PMC4486327

[26] Park J , Look KA. Health care expenditure burden of cancer care in the United States. Inquiry 2019;56:46958019880696. 31583928 10.1177/0046958019880696 PMC6778988

[27] Bernard DS , Farr SL, Fang Z. National estimates of out-of-pocket health care expenditure burdens among nonelderly adults with cancer: 2001 to 2008. J Clin Oncol 2011;29:2821–6. 21632508 10.1200/JCO.2010.33.0522 PMC3139395

[28] Deb P , Norton EC. Modeling health care expenditures and use. Annu Rev Public Health 2018;39:489–505. 29328879 10.1146/annurev-publhealth-040617-013517

[29] Williams R . Using the margins command to estimate and interpret adjusted predictions and marginal effects. Stata J 2012;12:308–31.

[30] Anderson MJ . Permutation tests for univariate or multivariate analysis of variance and regression. Can J Fish Aquat Sci 2001;58:626–39.

[31] Yabroff KR , Lund J, Kepka D, Mariotto A. Economic burden of cancer in the United States: estimates, projections, and future research. Cancer Epidemiol Biomarkers Prev 2011;20:2006–14. 21980008 10.1158/1055-9965.EPI-11-0650 PMC3191884

[32] Jacobson JO , Rotenstein LS, Berry LL. New diagnosis bundle: improving care delivery for patients with newly diagnosed cancer. J Oncol Pract 2016;12:404–6. 27048612 10.1200/JOP.2016.011163

[33] Chesney TR , Haas B, Coburn NG, Mahar AL, Zuk V, Zhao H, . Immediate and long-term health care support needs of older adults undergoing cancer surgery: a population-based analysis of postoperative homecare utilization. Ann Surg Oncol 2021;28:1298–310. 32789531 10.1245/s10434-020-08992-8

[34] Gotze H , Friedrich M, Taubenheim S, Dietz A, Lordick F, Mehnert A. Depression and anxiety in long-term survivors 5 and 10 years after cancer diagnosis. Support Care Cancer 2020;28:211–20. 31001695 10.1007/s00520-019-04805-1

[35] Pan X , Sambamoorthi U. Health care expenditures associated with depression in adults with cancer. J Community Support Oncol 2015;13:240–7. 26270540 10.12788/jcso.0150 PMC4576451

[36] Jeffery DD , Linton A. The impact of depression as a cancer comorbidity: rates, health care utilization, and associated costs. Community Oncol 2012;9:216–21.

[37] Cho O , Chun M. Management for locally advanced cervical cancer: new trends and controversial issues. Radiat Oncol J 2018;36:254–64. 30630264 10.3857/roj.2018.00500 PMC6361251

[38] Melamed A , Ramirez PT. Changing treatment landscape for early cervical cancer: outcomes reported with minimally invasive surgery compared with an open approach. Curr Opin Obstet Gynecol 2020;32:22–7. 31815768 10.1097/GCO.0000000000000598

[39] Blanco M , Chen L, Melamed A, Tergas AI, Khoury-Collado F, Hou JY, . Cost of care for the initial management of cervical cancer in women with commercial insurance. Am J Obstet Gynecol 2021;224:286 10.1016/j.ajog.2020.08.039 32818476

[40] Pfaendler KS , Wenzel L, Mechanic MB, Penner KR. Cervical cancer survivorship: long-term quality of life and social support. Clin Ther 2015;37:39–48. 25592090 10.1016/j.clinthera.2014.11.013 PMC4404405

[41] Donovan KA , Boyington AR, Judson PL, Wyman JF. Bladder and bowel symptoms in cervical and endometrial cancer survivors. Psychooncology 2014;23:672–8. 24481859 10.1002/pon.3461 PMC4043946

[42] Mirabeau-Beale KL , Viswanathan AN. Quality of life (QOL) in women treated for gynecologic malignancies with radiation therapy: a literature review of patient - reported outcomes. Gynecol Oncol 2014;134:403–9. 24844593 10.1016/j.ygyno.2014.05.008

[43] Osann K , Hsieh S, Nelson EL, Monk BJ, Chase D, Cella D, . Factors associated with poor quality of life among cervical cancer survivors: implications for clinical care and clinical trials. Gynecol Oncol 2014;135:266–72. 25192629 10.1016/j.ygyno.2014.08.036 PMC4479396

[44] Shaitelman SF , Cromwell KD, Rasmussen JC, Stout NL, Armer JM, Lasinski BB, . Recent progress in the treatment and prevention of cancer-related lymphedema. CA Cancer J Clin 2015;65:55–81. 25410402 10.3322/caac.21253 PMC4808814

[45] Vanneste BG , Van De Voorde L, de Ridder RJ, Van Limbergen EJ, Lambin P, van Lin EN. Chronic radiation proctitis: tricks to prevent and treat. Int J Colorectal Dis 2015;30:1293–303. 26198994 10.1007/s00384-015-2289-4 PMC4575375

[46] Keyes KM , Rutherford C, Popham F, Martins SS, Gray L. How healthy are survey respondents compared with the general population?: using survey-linked death records to compare mortality outcomes. Epidemiology 2018;29:299–307. 29389712 10.1097/EDE.0000000000000775 PMC5794231

[47] Althubaiti A . Information bias in health research: definition, pitfalls, and adjustment methods. J Multidiscip Healthc 2016;9:211–7. 27217764 10.2147/JMDH.S104807 PMC4862344

[48] Bethlehem J . Selection bias in web surveys. Int Statist Rev 2010;78:161–88.

[49] Szolnoki G , Hofmann D. Online face-to-face and telephone surveys—comparing different sampling methods in wine consumer research. Wine Econom Policy 2013;2:57–66.

[50] Zuvekas SH , Olin GL. Accuracy of Medicare expenditures in the medical expenditure panel survey. Inquiry 2009;46:92–108. 19489486 10.5034/inquiryjrnl_46.01.92

[51] Hill SC , Zuvekas SH, Zodet MW. Implications of the accuracy of MEPS prescription drug data for health services research. Inquiry 2011;48:242–59. 22235548 10.5034/inquiryjrnl_48.03.04

[52] Research AH and Quality. Medical expenditure panel survey (MEPS); 2021. Available from: https://www.ahrq.gov/data/meps.html.

[53] Ramsey SD , Bansal A, Fedorenko CR, Blough DK, Overstreet KA, Shankaran V, . Financial insolvency as a risk factor for early mortality among patients with cancer. J Clin Oncol 2016;34:980–6. 26811521 10.1200/JCO.2015.64.6620 PMC4933128

[54] Shankaran V , Ramsey S. Addressing the financial burden of cancer treatment: from copay to can't pay. JAMA Oncol 2015;1:273–4. 26181165 10.1001/jamaoncol.2015.0423

[55] Zafar SY , Newcomer LN, McCarthy J, Nasso SF, Saltz LB. How should we intervene on the financial toxicity of cancer care? One shot, four perspectives. Am Soc Clin Oncol Educ Book 2017;37:35–9. 28561659 10.1200/EDBK_174893

